# Touchdown General Primer (GP5+/GP6+) PCR and optimized sample DNA concentration support the sensitive detection of human papillomavirus

**DOI:** 10.1186/1472-6890-5-10

**Published:** 2005-11-16

**Authors:** Mark F Evans, Christine SC Adamson, Linda Simmons-Arnold, Kumarasen Cooper

**Affiliations:** 1 Department of Pathology, University of Vermont, Burlington, Vermont 05405, USA

## Abstract

**Background:**

The GP5+/GP6+ PCR assay is a well-established HPV detection technique. This study has examined the effects of incorporating 'hot start' and 'touchdown' steps into the protocol. In addition, dTTP was substituted with dUTP to permit contamination control measures against carry-over PCR product.

**Methods:**

Firstly, HPV-16 was amplified from SiHa cell DNA (0.1 ng–100 ng) diluted in a background of C-33A DNA (100 ng-2 μg). Secondly, the detection of small quantities (15ag-1.5pg) of HPV recombinant plasmids (types 16, 31, 33, 45, 51, 52, and 56) diluted in C-33A DNA was investigated. Thirdly, clinical sample DNA extracts (cervical smears, formalin-fixed vaginal lesions and breast tumors) were tested for HPV. Six different PCR protocols were assessed. HPV was detected by gel electrophoresis, and by Southern and dot blot hybridization.

**Results:**

HPV detection sensitivity was dependent on the total amount of DNA in a PCR. Touchdown protocols supported HPV-16 detection from 1 ng or 0.5 ng SiHa cell DNA in a background of 2 μg or 1 μg C-33A DNA respectively, and from 0.1 ng of SiHa cell DNA (~28 copies HPV-16) in 500 ng or 100 ng background DNA. Under standard GP5+/GP6+ annealing conditions, HPV-16 went undetected when the DNA content of a PCR was 2 μg or 1 μg, and with 500 ng C-33A DNA the sensitivity limit was 1 ng SiHa cell DNA. HPV recombinant plasmids were each detected with high (albeit varying) sensitivity by a touchdown protocol. HPV-31 was better amplified under standard annealing conditions (1.5fg in 100 ng background DNA) than by a touchdown approach (15fg detection limit). HPV-52 was not amplified by the standard protocol at the dilutions tested. Seventeen different HPV types were demonstrated in 47/65 (72%) abnormal cytology samples recorded as HPV negative by standard GP5+/GP6+ conditions. Twenty-one different HPV types were recorded in 111/114 (97%) vaginal lesions. Multiple infections were also detectable using a touchdown approach. Of 26 breast tumors, 5 (19%) tested HPV positive by the standard assay and 15/26 (58%) using a touchdown protocol.

**Conclusion:**

Touchdown modification of the GP5+/GP6+ PCR assay enables the detection of HPV undetected under regular assay conditions. The use of standardized DNA quantities in a PCR rather than standard sample volumes containing arbitrary amounts of DNA is supported. A touchdown approach may be beneficial as an analytical test for the re-evaluation of (apparently) HPV negative abnormal cervical cytological or histological samples, and for investigating the association of HPV with disease conditions at diverse organ sites. The clinical utility of a touchdown approach for HPV detection requires further investigation as increased assay analytical sensitivity may not necessarily equate with improved clinical sensitivity or specificity.

## Background

The association of human papillomaviruses (HPV) with invasive cervical carcinoma and its precursor lesions is well characterized [1,2]. There is also an emerging body of data indicating that HPV may contribute to tumor etiology at a variety of other anatomical sites [3]. For example, high-risk HPV types have been detected in up to 48% of breast carcinomas [4], although other studies have reported an absence of HPV in these tumors [5].

Clearly, any estimate of HPV prevalence amongst a tissue sample set is dependent on the detection method used. Commonly employed PCR based assays include the General Primer Mediated 5+/6+ (GP5+/GP6+) [6,7] and the MY09/MY11 [8] systems that amplify sequences from the *L1 *region of the HPV genome. Since the early/mid-90 s, when these assays were first developed a number of modifications that can improve PCR efficiency have been described. In addition, there have been improvements in thermal cycler specifications. This study has examined the effects of incorporating 'hot start' [9] and 'touchdown' [10] steps into the GP5+/GP6+ assay. Assays have been tested for use with dUTP instead of dTTP so that a uracil *N*-glycosylase (UNG) pre-PCR-incubation step can be included to degrade any contaminating carry-over PCR product present at reaction set up. The effect of the quantity of background DNA in an individual PCR on the limits of HPV detection has been specially investigated. Protocols have been tested on HPV recombinant plasmids, and DNA extracted from cervical cell lines, cervical cytology samples, and from formalin-fixed, paraffin-embedded (FFPE) vaginal intraepithelial neoplasia (VAIN) lesions and breast invasive ductal carcinomas (IDC).

## Methods

### Materials

All patient materials used in this study were obtained and analyzed with Institutional Review Board approval.

#### Cell lines

SiHa cells that contain one copy of the HPV-16 genome integrated at chromosome 13q21-31 [11], and C-33A cells derived from an HPV negative cervical carcinoma, were acquired from the American Tissue Culture Collection (ATCC), Manassas, VA.

#### HPV recombinant plasmids

HPV types 16, 45, and 51 were received courtesy of Dr. E-M de Villiers, Deutsches Krebsforschungszentrum (dkfz), Heidelberg, Germany. HPV-33 was received courtesy of Dr. Gerard Orth, Institut Pasteur, Paris, France. HPV-31, 52, and 56 were obtained from the ATCC.

#### Cervical cytology samples

Remnant cells (following cervical smear testing) were obtained from samples diagnosed as low-grade cervical squamous intraepithelial lesion (LSIL), abnormal squamous cells of undetermined significance (ASC-US), abnormal squamous cells cannot exclude HSIL (ASC-H), or, high-grade squamous intraepithelial lesion (HSIL).

#### Breast invasive ductal carcinomas

Twenty-six FFPE IDC samples were selected at random from Fletcher Allen Health Care Pathology (FAHC) archives.

#### Vaginal intraepithelial neoplasia samples

114 FFPE VAIN samples were retrieved from FAHC archives.

### DNA extraction and quantification

DNA was extracted and purified from cultured SiHa and C-33A cells and from cytology samples by proteinase K digestion followed with a column clean-up method (Qiagen *DN*easy Tissue Kit). DNA was extracted from FFPE tissues by digesting five 6 μm dewaxed sections with proteinase K as previously described [12]. DNA concentrations were estimated using a DyNA Quant 200 Fluorometer (Hoeffer Scientific).

### PCR protocols

The PCR protocols tested are summarized in Table 1. Initially, the standard GP5+/GP6+ assay cycling conditions and four different touchdown protocols were compared. In all protocols dTTP was substituted with dUTP (ACGU dNTP mix [Sigma A5593]) and included a pre-PCR incubation step with 'heat-killed'-Uracil *N*-Glycosylase (HK™-UNG, Epicentre). HK™-UNG is a heat-labile form of UNG that has maximal activity at 50°C and is designed to be completely inactivated by 10 minute incubation at 65°C (Epicentre technical note). Samples were incubated at 37°C for 30 min with 0.2U HK™-UNG according to supplier's recommendations. A 'hot-start' step followed utilizing HotStarTaq DNA Polymerase (Qiagen), which requires incubation at 95°C for 15 min to activate the Taq. HotStarTaq was used at a concentration of 1U per 50 μl reaction. Magnesium chloride was included in reactions at a concentration of 4 mM. Other reaction conditions held constant for each protocol included 1X HotStarTaq buffer [Tris-HCl (pH8.7), KCl, (NH_4_)_2_S0_4_], and each primer at a concentration of 1 μM, (GP5+ [5'-TTTGTTACTGTGGTAGATACTAC-3'] and GP6+ [5'-GAAAAATAAACTGTAAATCATATTC-3']). The *T*_m _of the GP5+ primer is 45°C and the *T*_m _of the GP6+ primer is 41°C, (*T*_m _values are estimated for 50 mM salt conditions and are calculated from the equation *T*_m_= 59.9 + 41 [%GC] - [675/Primer Length]).

**Table 1:** Details of the PCR protocols tested. All protocols included substitution of dTTP with dUTP and commenced with a uracil *N*-glycosylase (UNG) incubation step (37°C for 30 minutes) followed by a HotStarTaq DNA Polymerase (Qiagen) activation/UNG inactivation step (95°C, 15 min).

**Protocol**	**Denaturation**	**Touchdown annealing cycles**	**Additional annealing cycles**	**Extension**	**Final extension**
**GP5+/GP6+**	1 min, 94°C	-	2 min, 40°C (40–50 cycles)	1.5 min, 72°C	4 min, 72°C
**TDP1**	1 min, 94°C	2 min, 45°C to 40°C in 0.5°C decrements (11 cycles)	2 min, 40°C (29–39 cycles)	1.5 min, 72°C	4 min, 72°C
**TDP2**	1 min, 94°C	2 min, 50°C to 40°C in 1.0°C decrements (11 cycles)	2 min, 40°C (29–39 cycles)	1.5 min, 72°C	4 min, 72°C
**TDP3**	1 min, 94°C	2 min, 50°C to 40°C in 0.5°C decrements (21 cycles)	2 min, 40°C (19–29 cycles)	1.5 min, 72°C	4 min, 72°C
**TDP4**	1 min, 94°C	2 min, 55°C to 40°C in 1.0°C decrements (16 cycles)	2 min, 40°C (24–34 cycles)	1.5 min, 72°C	4 min, 72°C

The standard GP5+/GP6+ amplification cycles comprise 1 min at 94°C, 2 min at 40°C, and 1.5 min at 72°C, with the final extension step prolonged to 4 min to ensure complete amplification of the 140–150 base pair products [6,7]. The standard denaturation and extension temperatures and times were retained in the four 'touchdown' protocols tested. The annealing period of 2 min was also retained, as was the 'final' annealing temperature of 40°C. Touchdown protocols (Table 1) with starting annealing temperatures of 45°C, 50°C, or 55°C, decreasing by 0.5°C or 1.0°C decrements per PCR cycle down to 40°C were evaluated. All PCR protocols were tried at 40 up to 50 cycles of amplification. Protocols were variously tested with 5 ng up to 2 μg of DNA per 50 μl reaction. Experiments were conducted using 0.2 ml PCR tubes (lightly coated with mineral oil to enhance heat conduction) and a Programmable Thermal Controller-100™ [PTC-100] (MJ Research, Inc. MA).

Following completion of the study the authors became aware of a GP5+/GP6+ protocol incorporating modified ramping times (MRT) [13]. The MRT GP5+/GP6+ conditions were tested on a subset of the samples. For the purposes of this study, the protocol was modified to include hot-start, dUTP, and 50 PCR cycles. Reaction ingredients were as above. The MRT GP5+/GP6+ cycling conditions were as follows:

HK™-UNG incubation step: 37°C 30 mins

Denaturation/HotStarTaq activation: 95°C 15 mins

Cycles (n = 50) 20 s at 94°C

In 24 s to 90°C

In 66 s to 48°C

In 30 s to 38°C

30 s at 38°C

In 18 s to 42°C

In 42 s to 71°C

80 s at 71°C

In 24 s to 69°C

In 90 s to 94°C

Final step: 4 min at 71°C

### Control measures

#### PCR

Negative control PCR was performed using C-33A cells and reactions containing no template DNA. PCR amplification of β-globin sequences was performed to confirm sample fitness for PCR assay [14].

#### FFPE tissue-block sectioning

Measures to prevent potential cross-contamination of tissue during sample sectioning, included wiping the microtome blade with histoclear (xylene substitute) and 'DNA-Erase' [ICN] (a DNA contamination removal reagent) between blocks. Additionally, the first 10–20 sections cut from a specimen were discarded prior to collecting sections for DNA extraction from the specimen.

### HPV detection

Protocol sensitivity was measured by the presence of a ~150 base-pair band after 10 μl PCR product had been sieved through a 2.5% agarose gel stained with 0.5 μg/ml ethidium bromide. Southern blot hybridization was performed to test that the ~150 base-pair PCR product represented HPV DNA. HPV typing was performed by dot blot hybridization of PCR products with up to 37 type-specific biotin-labeled oligonucleotide probes. Biotin was detected with streptavidin-alkaline phosphatase conjugate and the substrate nitrobluetetrazolium/5-bromo-4-chloro-3-indolyl phosphate [12], or by sequencing.

### Protocol reliability

Touchdown protocols were assessed on two or more occasions on SiHa cell/C-33A DNA dilutions, and the TDP3 was also tested several times on recombinant HPV plasmid samples to determine data reproducibility. Clinical samples (cytology, FFPE VAIN and breast tumors) were tested once only with PCR protocols. Inter-laboratory tests of different PCR assays were not performed.

## Results

### Detection of HPV in SiHa cells

**Figure 1:**
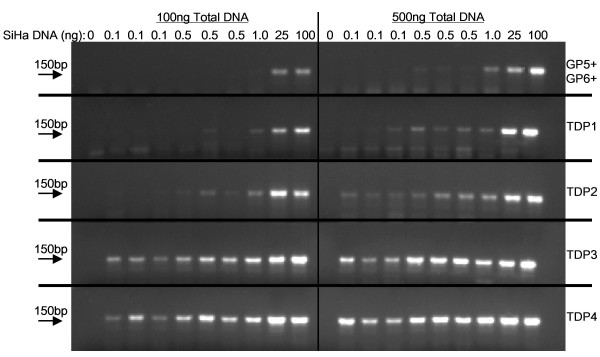
**Detection of HPV-16 PCR product by agarose gel electrophoresis**. SiHa cell DNA, 0, 0.1, 0.5, 1.0, 25, or 100 ng, was diluted in C-33A DNA to give a total of 0.5 μg or 0.1 μg DNA. The GP5+/GP6+, TDP1, TDP2, TDP3 and TDP4 assays were compared for the detection of HPV-16 product on an ethidium bromide (0.5 μg/ml) stained 2.5% agarose gel. RB: reagent blank, M: 50 base pair molecular weight ladder.

**Figure 2:**
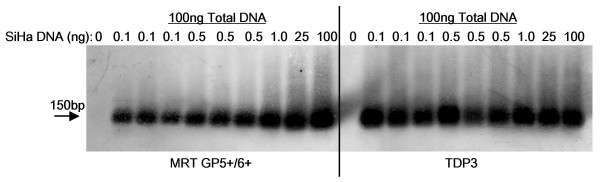
**Southern blot of TDP3 and MRT GP5+/GP6+ PCR products**. SiHa cell DNA, 0, 0.1, 0.5, 1.0, 25, or 100 ng, was diluted into a total of 0.1 μg DNA made up with C-33A DNA. After PCR with the TDP3 and MRT protocols and gel electrophoresis, products were electroblotted onto nylon membrane and hybridized with biotin-labeled HPV-16 oligonucleotide probe detected with streptavidin-alkaline phosphatase and NBT/BCIP. The data confirm the ~150 bp amplicons represent HPV sequences.

The ability of the protocols to amplify HPV-16 from 100 ng, 25 ng, 1 ng, 0.5 ng or 0.1 ng of SiHa cell DNA 'hidden' in a total quantity of either 2 μg, 1 μg, 500 ng or 100 ng DNA (made up with C-33A DNA) was examined. The results are summarized in Table 2, and Figure 1. Each touchdown protocol improved the detection of HPV sequences. The best protocols were the TDP3 and TDP4 that demonstrated HPV-16 amplification from 0.1 ng of SiHa cell DNA in a total DNA content of 100 ng or 500 ng (Figure 1). When the total DNA content was 1 μg, these protocols enabled successful HPV-16 amplification from 0.5 ng of SiHa DNA (Table 2). HPV-16 was amplifiable by the TDP3 protocol from 1 ng of SiHa cell DNA when the total reaction DNA was 2 μg (Table 2). The least successful protocol was the standard GP5+/GP6+ that did not amplify HPV-16 from SiHa cell DNA diluted in 1 μg or 2 μg of background DNA and was only able to demonstrate HPV-16 from 1 ng of SiHa cell DNA contained in a total of 500 ng DNA (Table 2, Figure 1). Data was confirmed by repeat PCR tests. The MRT GP5+/GP6+ protocol was also tested on SiHa cell DNA diluted in a background of 100 ng C-33A DNA and supported detection of HPV-16 from 0.1 ng SiHa cell DNA. Figure 2 details a Southern blot of PCR products from the TDP3 and MRT protocols hybridized with a biotin-labeled HPV-16 oligonucleotide probe.

**Table 2:** PCR protocol sensitivities for the detection of HPV-16 in SiHa/C-33A DNA mixtures. SiHa cell DNA (0.1 ng to 100 ng) was mixed with C-33A cell DNA to give final DNA quantities of 2 μg, 1 μg, 0.5 μg, or 0.1 μg. These preparations were tested with up to six different PCR protocols. HPV-16 detection was most successful using by the TDP3 and TDP4 conditions. Data are for protocols tested at 50 PCR cycles.  HPV detected,  HPV undetected, ND: not done.

	**Total DNA in PCR: 2 μg**					**Total DNA in PCR: 1 μg**				
**SiHa DNA**	**100 ng**	**25 ng**	**1 ng**	**0.5 ng**	**0.1 ng**	**100 ng**	**25 ng**	**1 ng**	**0.5 ng**	**0.1 ng**

**GP5+/6+**										
**TDP1**										
**TDP2**										
**TDP3**										
**TDP4**	ND	ND	ND	ND	ND					
**MRT**	ND	ND	ND	ND	ND	ND	ND	ND	ND	ND

	**Total DNA in PCR: 0.5 μg**					**Total DNA in PCR: 0.1 μg**				

**SiHa DNA**	**100 ng**	**25 ng**	**1 ng**	**0.5 ng**	**0.1 ng**	**100 ng**	**25 ng**	**1 ng**	**0.5 ng**	**0.1 ng**

**GP5+/6+**										
**TDP1**										
**TDP2**										
**TDP3**										
**TDP4**										
**MRT**	ND	ND	ND	ND	ND					

### Detection of HPV recombinant plasmid DNA

**Figure 3:**
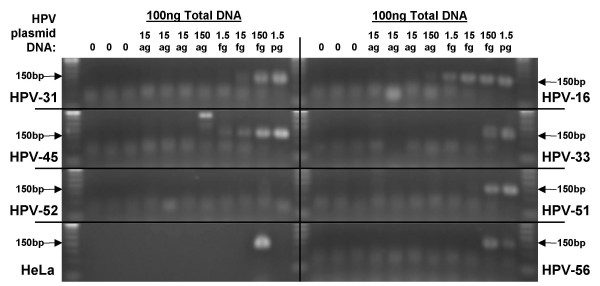
**Detection of recombinant HPV plasmid by GP5+/GP6+ PCR**. HPV (types 16, 31, 33, 45, 51, 52, and 56) recombinant plasmids, 0, 15ag, 150ag, 1.5fg, 15fg, 150fg, 1.5pg, were diluted in 100 ng C-33A DNA and amplified (50 cycles) using the GP5+/GP6+ protocol. Positive control amplification of HPV-18 from 100 ng HeLa cell DNA is also shown.

**Figure 4:**
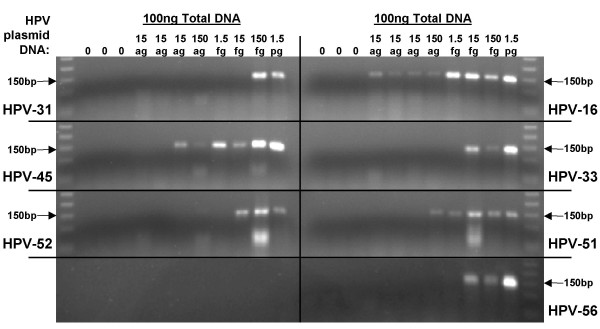
**Detection of recombinant HPV plasmid by TDP3 PCR**. HPV (types 16, 31, 33, 45, 51, 52, and 56) recombinant plasmids, 0, 15ag, 150ag, 1.5fg, 15fg, 150fg, 1.5pg, were diluted in 100 ng C-33A DNA and amplified (50 cycles) using the TDP3 conditions.

The ability of the standard GP5+/GP6+ and TDP3 protocols (50 cycles each) to amplify 15ag, 150ag, 1.5fg, 15fg, 150fg, or 1.5pg of HPV-16, 31, 33, 45, 51, 52, or, 56 plasmid in a background of C-33A cell DNA (to a total of 100 ng DNA per PCR) was examined. The same set of 'master dilutions' were used in the two different PCR assays. The results are shown in Figures 3 and 4, and Table 3. With the TDP3 protocol HPV-16 amplification product was observed from (all three) 15ag recombinant plasmid dilutions, and HPV-45 from one of three 15ag HPV DNA dilutions. HPV-51 was detectable following amplification from 150ag DNA template; HPV-33, 52, and 56 from 15fg template; and, HPV-31 from 150fg recombinant HPV DNA. With the GP5+/GP6+ protocol HPV-16 was amplifiable from 150ag recombinant plasmid; HPV-45 from 1.5 g template; HPV-31 from 15fg; and HPV-33, 51, and 56 from 150fg template; HPV-52 was not detected. Data were confirmed up on repeat PCR.

**Table 3:** PCR sensitivities for the detection of seven different HPV types. HPV recombinant plasmids were diluted in a background of 100 ng C-33A DNA and amplified (50 cycles) by the GP5+/GP6+ protocol or by the TDP3 method.  HPV detected,  HPV undetected, **Σm **Total number of mismatches between the GP5+/GP6+ primers and the target HPV type.

		**GP5+/GP6+**						**TDP3**					
**Σm**	**HPV**	**1.5pg**	**150fg**	**15fg**	**1.5fg**	**150ag**	**15ag**	**1.5pg**	**150fg**	**15fg**	**1.5fg**	**150ag**	**15ag**

2	16												
3	33												
3	45												
4	31												
4	56												
7	52												
10	51												

### Detection of multiple HPV types

**Figure 5:**
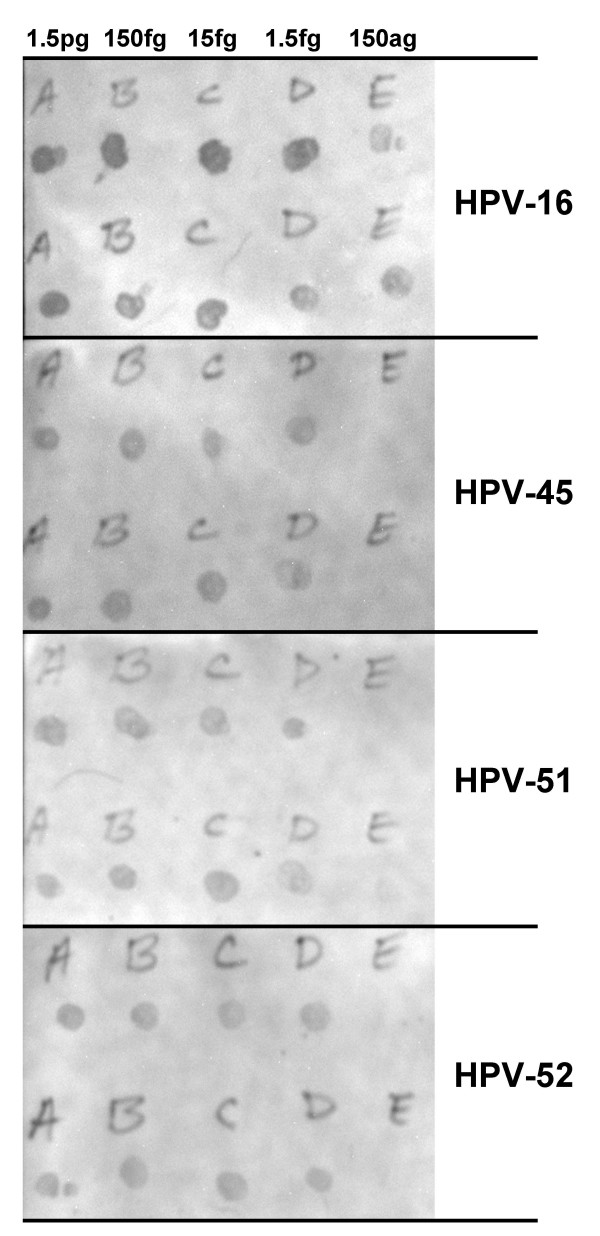
**Dot blot hybridization detection of multiple HPV types after TDP3 PCR**. HPV types 16, 45, 51, and 52 were combined (1.5pg, 150fg, 15fg, 1.5fg, or 150ag of each type) in a background of 100 ng C-33A DNA. After PCR, 0.6 μl of denatured product was spotted onto nylon membrane and hybridized with biotin-labeled HPV type specific oligonucleotide probe. Duplicate dot preparations were prepared.

To examine how a touchdown protocol affected the detection of multiple HPV types, dilutions of HPV-16, 45, 51, and 52 were combined in a background of 100 ng C-33A DNA. Five mixtures were compared: 1.5pg each HPV type combined in a PCR, 150fg of each type in a PCR, 15fg of each type in a PCR, 1.5fg of each type in a PCR, or 150ag of each type in a PCR. Following amplification using the TDP3 protocol, duplicate rows of 0.6 μl heat-denatured product was dot-blot hybridized with type-specific probes. The results are shown in Figure 5. HPV-16 was detectable following amplification of each dilution mixture. HPV types 45, 51, and 52 were detectable following amplification of mixtures containing 1.5pg, 150fg, 15fg, or 1.5fg, but not 150ag HPV DNA. Repeat PCR tests were not performed.

### Detection of HPV in cytology samples

**Figure 6:**
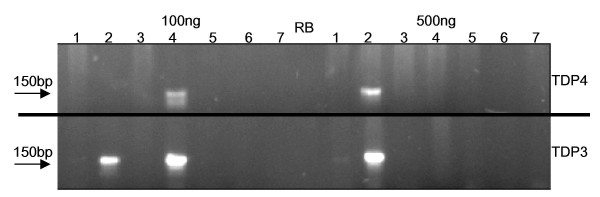
**Amplification of HPV from abnormal cervical cell DNA samples by TDP3 and TDP4**. Seven samples that tested negative for HPV using standard GP5+/GP6+ cycling conditions were assessed with the TDP3 and TDP4 assays. Both TDP3 and TDP4 supported the detection of HPV in sample 4 when 100 ng DNA was subject to PCR but not when 500 ng DNA was in a reaction. HPV was detected in sample 2 by the TDP3 protocol with 100 ng or 500 ng template DNA and by the TDP4 protocol at 500 ng but not 100 ng of sample. RB: reagent blank.

**Figure 7:**
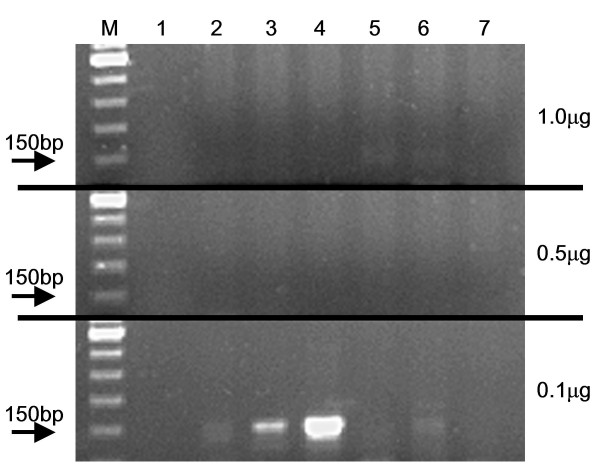
**Detection of HPV by TDP3 in abnormal cervical cell samples**. Samples had previously tested negative for HPV using standard GP5+/GP6+ cycling conditions. HPV detection was dependent on the total amount of DNA included in the PCR.

In a previous study [15], GP5+/GP6+ PCR was used to determine HPV type and prevalence amongst a set of abnormal cytology samples and 290/355 (82%) were recorded as HPV positive. The TDP3 and TDP4 protocols were tested on 100 ng and 500 ng of DNA from samples that had tested HPV negative. Both protocols detected HPV in samples previously recorded as HPV negative, with the TDP3 representing a more efficient assay (Figure 6). In some instances, HPV sequences were only detectable when 100 ng of DNA was included in the PCR, and samples were apparently HPV negative when 1.0 μg or 500 ng of sample DNA was used in an assay (Figure 7). All 65 abnormal cytology samples previously recorded as HPV negative were repeated with 50 cycles of the TDP3 protocol using 100 ng of sample DNA as template and 47 (72%) samples were recorded as HPV positive. Seventeen HPV types (6, 11, 16, 18, 31, 33, 35, 40, 42, 52, 58, 66, 67, 73, 81 [CP8304], 84 [MM8], and cand91 [CP6108]) were detected amongst these 47 samples. Multiple infections were recorded in 9/47 (19%) samples.

Fifty-four of the 65 'HPV negative' samples were also tested with the MRT protocol. Of these 54 samples, 10 were HPV negative and 44 HPV positive with the TDP3 protocol. Of the 10 negative samples, 1 tested HPV-53 positive by the MRT method, and of the 44 positive samples, 16 (36%) were negative with the MRT method. Five HPV types (16, 18, 31, 35, and 81) went undetected by the MRT method in this sample. An additional 25 samples that were negative following primary screening by the TDP3 assay were identified; none tested HPV positive after PCR with the standard GP5+/GP6+ protocol (50 PCR cycles). Two of the 25 samples tested positive (for HPV-39, and for HPV-51) by the MRT protocol. Thus, of 44 samples negative with the standard GP5+/GP6+ assay, 44 (100%) tested positive with the TDP3 protocol, and 28 (64%) tested positive with the MRT conditions; and, of 35 samples negative by the standard GP5+/GP6+ assay and the TDP3 assay, 3 (8.6%) tested positive with the MRT protocol.

The TDP3 protocol has subsequently been tested on 799 abnormal cytological samples and 752 (94%) have tested positive for one or other of 37 types: HPV-6, 11, 16, 18, 31, 32, 33, 35, 39, 40, 42, 43, 44, 45, 51, 52, 54, 55, 56, 58, 59, 61, 62, 66, 67, 69, 70, 72, 73, 81, 82, 83, 84, 87, cand89, cand90, and cand91.

### Detection of HPV in FFPE VAIN samples

**Figure 8:**
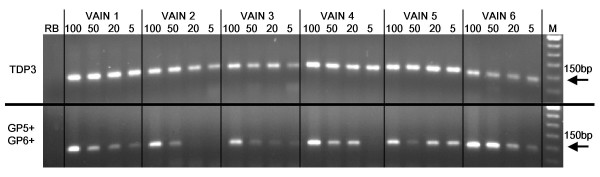
**HPV detection in six VAIN lesions by GP5+/GP6+ and TDP3 assays**. Sample DNA in PCR: 100 ng, 50 ng, 20 ng, or 5 ng. M: 50 bp molecular weight marker, RB: reagent blank.

The GP5+/GP6+ and TDP3 protocols (both 50 cycles) were compared for the detection of HPV in 5 ng, 20 ng, 50 ng and 100 ng of DNA extracted from six VAIN lesions (Figure 8). HPV sequences were detectable by TDP3 assay in all six samples at each sample DNA quantity. The GP5+/6+ assay detected HPV in all six cases, but electrophoretic band strength was strong only with 100 ng DNA in the PCR and tended to be weaker or absent at other template DNA quantities (Figure 8). Subsequent analysis (using the TDP3 conditions) of VAIN lesions from 114 patients demonstrated HPV in 111 (97.4%) samples. Twenty-one HPV types were identified: HPV types 6, 11, 16, 18, 31, 33, 35, 40, 42, 43, 45, 51, 54, 56, 58, 59, 73, 81, 83, 84, and cand90. Double or multiple infections were detected in 19 (13%) samples.

### Detection of HPV in FFPE breast invasive ductal carcinoma samples

**Figure 9:**
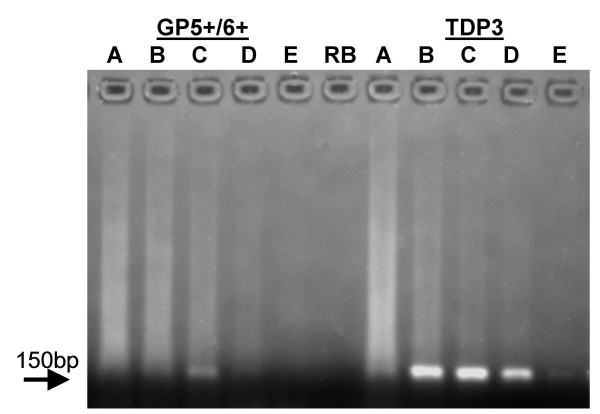
**Comparison of GP5+/GP6+ and TDP3 amplification of HPV sequences from a FFPE breast invasive ductal carcinoma**. Sample DNA in PCR, A: 450 ng, B: 325 ng, C: 225 ng, D: 100 ng, E: 20 ng. The TDP3 assay facilitated detection of HPV in 100 ng, 225 ng and 325 ng sample, whereas with GP5+/6+ assay conditions a HPV was detectable (weak band) only when 225 ng sample was in the PCR. 50 cycles of PCR were used with each of the protocols. RB: reagent blank.

**Figure 10:**
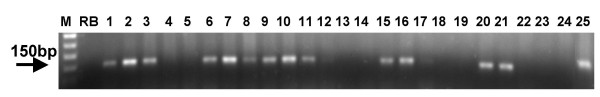
**Demonstration of HPV in FFPE breast invasive ductal carcinomas following TDP3 amplification**. 200 ng of sample DNA were subject to a total of 50 PCR cycles. Samples 1, 2, 3, 6, 7, 8, 9, 10, 11, 15, 16, 20, 21 and 25 were recorded as HPV positive following electrophoresis and dot blot hybridization. M: 50 bp molecular weight marker, RB: reagent blank.

A preliminary assay of 26 IDC samples (arbitrary 10 μl of DNA extract) by GP5+/GP6+ PCR demonstrated faint bands upon electrophoresis for five (19%) samples. Following DNA quantification, one of these samples was compared for detection of HPV by GP5+/GP6+ assay and TDP3 assay (both for 50 cycles), using 20 ng, 100 ng, 225 ng, 325 ng or 450 ng of DNA extract in the PCR (Figure 9). A weak band was obtained using the GP5+/GP6+ assay following amplification from 225 ng DNA template but HPV was not detected at any of the other sample concentrations. Bands were clearly visible using the TDP3 assay from 100 ng, 225 ng and 325 ng sample quantities. There is a faint band after amplification from 20 ng, but amplification is not evident for 450 ng sample DNA. The TDP3 assay was subsequently applied to the other 25 breast tumor samples (~200 ng sample DNA in the PCR) and HPV was detected in another 14 instances (Figure 10). Overall 15/26 (58%) breast tumors tested positive by this assay for HPV. Dot blot hybridization demonstrated fourteen samples were positive for HPV-16 and one for HPV-31.

## Discussion

The main finding of this study is that touchdown general primer PCR coupled with control of the quantity of sample DNA in a PCR supports the detection of low-copy number HPV in an excess background of human DNA sequences. A high percentage of abnormal cervical cytological samples previously recorded as HPV negative tested HPV positive by this approach. It has also been demonstrated that a wide range of HPV types and multiple infections are detectable using a touchdown protocol.

### Detection of minority HPV DNA

The potential of PCR to detect minority nucleic acid species is the genius of the technique. However, the presence of background DNA may compromise PCR efficiency by giving rise to non-specific primer annealing [16]. Assays such as the GP5+/GP6+ use one pair of primers to amplify a wide range of HPV types and necessarily each primer contains varying numbers of mismatches relative to any given HPV type [17]. Low-stringency primer-annealing conditions were originally defined for the GP5+/GP6+ assay [7] however, these conditions permit GP5+/GP6+ primer annealing to human sequences [6].

In this study a hot start step was introduced to prevent annealing that can occur at PCR set-up between primers and single-stranded DNA sequences produced during DNA extraction [9,16]. Four touchdown protocols were tested to assess how a touchdown annealing approach affects the amplification of HPV sequences. In the first instance, these protocols were tested on a model system consisting of small quantities of SiHa cell DNA 'hidden' in varying amounts of C-33A cell DNA.

The SiHa cell line is known to contain approximately one slightly truncated copy of the HPV-16 genome integrated in chromosome 13q21-31 [11]. The cell line is hyper-diploid but has been shown to be disomic with respect to chromosome 13 [11]. It is possible to approximate the number of copies of HPV-16 detected by the various protocols tested assuming for the purpose of the approximation that there are 6.6 × 10^9 ^DNA base pairs per SiHa cell. One base pair weighs 650 daltons and one dalton weighs 1.66 × 10^-24^g. Therefore, one diploid cell contains 7.12pg of DNA, and there will be one copy of the GP5+/GP6+ primer target per 3.56pg of SiHa cell DNA extract. It follows that the number of copies of HPV-16 in 0.1 ng of SiHa cell DNA will be ~28; in 0.5 ng SiHa DNA there will be ~140 copies; in 1 ng ~280 copies; in 25 ng ~7000 copies and in 100 ng ~28,000. HPV-16 was amplifiable from smaller quantities of SiHa cell DNA as the total quantity of DNA reduced from 2 μg to 500 ng for all protocols. Two touchdown protocols (TDP3, TDP4) were routinely able to detect 0.1 ng SiHa HPV DNA (~28 copies) in a total DNA quantity of 500 ng (approximately equal to DNA extracted from 70,000 cells [i.e. 1 copy HPV-16 detected per 2500 cells]). In contrast, the best performance of the GP5+/GP6+ protocol was the demonstration of HPV-16 from 1 ng of SiHa cell DNA (~280 HPV-16 copies) in a background of 500 ng total DNA. Unlike TDP3 and TDP4 protocols, the standard GP5+/GP6+ assay failed to demonstrate HPV when the total DNA content in a reaction was 1 μg or 2 μg. It is noticeable from Figure 1 that some of the protocols gave slightly more intense bands, ~150 bp in size, following amplification of HPV-16 sequences in a background of 500 ng DNA compared to the amplification in a background of 100 ng. This empirical finding was reproducible on repeat PCR. This observation is counter-intuitive; however, given the nature of the touchdown approach to annealing, the character of the dilutions, and the high number of PCR cycles, the PCR assays tested are essentially qualitative rather than quantitative.

The significantly improved detection of low-copy HPV-16 by touchdown protocols illustrates the important influence background DNA can have on detection sensitivity. The findings are particular noteworthy given that relative to the target HPV-16 sequence, the GP5+ primer sequence (*T*_m _= 45°C) has just two mismatches and the GP6+ primer (*T*_m _= 41°C) has no mismatches. The data show that under low-stringency annealing conditions and when the target is low-copy HPV, considerable non-specific primer annealing with human sequences must occur despite the near perfect homology of the primers for the HPV-16 *L1 *open reading frame target.

The MRT GP5+/GP6+ protocol was also found to detect low-copy HPV in a background of human sequences. This assay incorporates periods of slow temperature changes from the denaturation step to the annealing step and from the annealing step to the extension step. This approach may allow for better specific annealing of primers with HPV target during the gradual cooling to the final annealing temperature of 38°C than is possible with the standard conditions. Possibly, the express heating/cooling default settings of current generation thermal cyclers may compromise the efficiency of the GP5+/GP6+ PCR assay as developed using early generation machines [6,7].

### Detection of HPV recombinant plasmid DNA

To examine the effect of a touchdown protocol on a wider range of HPV types, recombinant HPV/plasmid DNA samples were diluted in C-33A DNA and tested with the TDP3 protocol. There are three primer mismatches with respect to HPV-33, one in the GP5+ primer and two in the GP6+ primer. For HPV-45 there are three mismatches that are all in the GP5+ primer sequence. HPV-31 and 56 each have one mismatch in the GP5+ primer and three in the GP6+ primer. For HPV-52 there are seven mismatches, including five in the GP5+ primer. HPV-51 has the greatest number of mismatches of any HPV type amplified by the GP5+/6+ system: six mismatches in the GP5+ primer and four in the GP6+ primer. Not surprisingly, HPV-16 was the most efficiently amplified type (Figure 3). Amplification product was detectable from 15ag DNA (approximately equivalent to 1 copy of the recombinant HPV plasmid) in a background of 100 ng human DNA (equivalent to 14,000 cells). HPV-45 and 51 were amplifiable from 150ag (10 HPV/plasmid copies); types 33, 52, and 56 from 15fg (100 copies of HPV) DNA; and, HPV-31 from 150fg (1000 copies) of recombinant DNA. Clearly, for such small quantities of DNA, slight variations in pipetting accuracy and/or DNA concentration estimate might alter the final sensitivity data. Nonetheless, the data indicates firstly that a touchdown protocol can sensitively amplify a range of HPV types, and secondly that the efficiency of amplification may not be a simple function of the number of mismatches between the primer and target sequence. With the TDP3 protocol HPV-31 (4 mismatches) was less efficiently amplified than HPV-56 (4 mismatches), HPV-52 (7 mismatches), or HPV-51 (10 mismatches) (Figure 4, Table 3). The TDP3 amplified six of the seven HPV types tested with greater sensitivity than the standard GP5+/GP6+ conditions. HPV-31 was detectable as a faint band from 15fg template DNA following the standard PCR, whereas the detection limit with the TDP3 protocol was 150fg (Figures 3 and 4, Table 3). HPV-52 was not detectable by the standard protocol at the dilution range tested but was detectable from 15fg starting template using the TDP3 protocol. These data again indicate that the detection efficiency for different HPV types by a general primer method may not follow easily predictable rules. General primer annealing involves a dynamic inter-relationship depending on HPV copy number, primer affinity for different HPV types, human sequence concentration, and, primer affinity for human sequences at a given annealing temperature. The empirical data indicate that a touchdown annealing approach helps negotiate these variables to support sensitive detection of a wide range of HPV types.

### Detection of multiple HPV types

This study also examined the ability of a touchdown protocol to amplify HPV types-16 (2 mismatches), 45 (3 mismatches), 51 (10 mismatches), and, 52 (7 mismatches) combined in one PCR (Figure 5). Again, HPV-16 was detected with the highest sensitivity; however, the other three types were identifiable following dot blot hybridization demonstrating that the touchdown protocol did not favor HPV-16 amplification to the exclusion of other types. An exhaustive assessment of the effect of different concentrations of HPV types within a PCR on the detection of multiple types was beyond the resources available for this study.

### Detection of HPV in cervical smear samples

An excess of background human DNA is likely to be a common situation when performing PCR on DNA extracted from routine cervical smear samples. The extent to which normal or lesion tissues are scraped from the cervix cannot be controlled. For efficient HPV PCR amplification, the study data strongly support the use of standardized quantities of DNA and not regular volumes containing arbitrary amounts of DNA. Overall, the cell line and clinical sample studies indicate that in a 50 μl reaction, between 100 ng and 200 ng sample DNA supports the most efficient PCR. Using optimized amounts of DNA, the TDP3 protocol demonstrated one or other of seventeen different HPV types in 47 of 65 (72%) abnormal cytology samples HPV negative by the regular GP5+/GP6+ protocol. Multiple infections were found in 9/47 (19%) of the samples.

The MRT GP5+/GP6+ conditions were tested on 54 of the 65 GP5+/GP6+ negative samples. Twenty-eight (64%) of 44 samples positive by the TDP3 were also positive by the MRT protocol, and 1 of 10 samples negative by the TDP3 protocol was positive by the MRT. Of an additional 25 abnormal cytological samples negative for HPV by the TDP3 assay, none tested positive with the standard GP5+/GP6+ method, but two samples were positive by the MRT protocol. Again, these data show that HPV detection can be highly dependent on assay conditions and also demonstrate that two or more methods may be required for inclusive HPV screening. Nevertheless, the above comparison of methods together with the demonstration of HPV in 94% of 799 abnormal cytological samples indicates a touchdown approach supports wide-ranging and sensitive HPV detection.

### Detection of HPV in FFPE samples

This study also examined the effect of touchdown PCR and sample DNA quantity on the amplification of HPV from FFPE tissues, where DNA quality is likely to be poor. VAIN lesions were chosen as an example of tissues from which HPV virions are actively shed and therefore where HPV DNA is in a relative abundance [18]. Amplification of HPV from VAIN lesions was readily accomplished by both the GP5+/GP6+ and TDP3 protocols although the TDP3 assay was able to amplify HPV from smaller quantities of template DNA than the GP5+/GP6+ assay (Figure 8). The data again illustrates the effect of DNA content on amplification efficiency. Assay of 114 VAIN lesions by TDP3 has demonstrated 21 different HPV types in 111 HPV positive samples, with double or multiple infections in 17 (15%) of the samples, again suggesting that a touchdown approach sensitively detects a wide range of HPV types.

Breast carcinomas were chosen for study as an example of a tumor where there are widely differing estimates of HPV prevalence. Most early studies of breast tumors found no evidence of HPV following PCR assay [5,19-21]. However, other investigations (by Southern blot hybridization, and by PCR) have reported high-risk HPV types (e.g. HPV-16, 18, or 33) in 24/50 (48%), 5/17 (30%), 19/41 (46%), 14/32 (44%), and 6/17 (35%) breast carcinomas [4,22-25]. de Villiers *et al*. PCR tested paired breast carcinoma and nipple tissues from 29 patients for anogenital and skin HPV types. HPV was detected in 25/29 (86%) carcinomas and in 20/29 (69%) nipple tissues [26]. In the present study, application of the GP5+/GP6+ assay using an arbitrary DNA quantity in the PCR indicated 5/26 (19%) invasive ductal carcinomas were HPV positive. Subsequent assays using the TDP3 protocol and 200 ng of sample indicated 15/26 (58%) tumors were HPV positive. These data add to the evidence that HPV sequences are present in breast tumor tissues. However, that HPV was detectable in most tumors only after use of a highly optimized PCR protocol may indicate that HPV is in a latent form and/or is confined to a subset of tumor cells or other cells associated with a tumor mass. Perhaps there may be parallels with Epstein Barr Virus (EBV) detection in breast tumors. EBV has been reported in up to 51% of breast carcinomas by sensitive PCR assay [27], but it has since been suggested the EBV detected is amplified from EBV positive tumor infiltrating lymphocytes and not from tumor cells *per se *[28]. Further studies are required to determine whether HPV detected in breast tumors represents an incidental 'passenger' or has causal significance. Additionally, given the disparate estimates of HPV prevalence in breast tumors, future studies might incorporate more rigorous control measures such as cutting sections of HPV negative tissues for DNA extraction and PCR in between successive breast tumor specimens to test for cross-contamination with HPV positive samples.

### Carry-over contamination control

In this study dTTP was replaced with dUTP in all protocols in order to identify a sensitive PCR test that also includes a control measure against carry-over contamination. However, uracil *N*-glycosylase (UNG) is an enzyme that has a degree of thermostability and may also become reactivated after completion of a PCR protocol [29,30]. HK™-UNG (Epicentre) is designed to be heat-labile and free of such shortcomings, but it has been reported that even heat-labile forms of UNG may retain some residual activity and spoil PCR efficiency [29]. Further, dUTP is less efficiently incorporated into PCR amplicons than dTTP [30]. UNG/dUTP usage might therefore result in suboptimal PCR assay performance and even greater sensitivity for HPV might be possible for all the protocols tested using regular dNTPs.

### Clinical utility

The impetus for this study was to determine whether abnormal cytological samples that tested HPV negative by a commonly used PCR assay were in fact HPV positive. Using a touchdown protocol, HPV has been demonstrated in a high percentage of samples previously recorded as HPV negative consistent with HPV representing a necessary cause of most (if not all) abnormal cervical cytological conditions. However, an assay with high analytical sensitivity for HPV might not be appropriate as a clinical test. The HPV status of cytological samples has been proposed as a marker to identify patients with underlying high-grade cervical intraepithelial neoplasia (CIN) [31] and current clinically approved HPV tests detect the bulk of high-grade CIN and invasive cervical carcinoma. Increased sensitivity for HPV might lead to reduced test specificity for high-grade CIN.

## Conclusion

This study has demonstrated that a touchdown modification of the GP5+/GP6+ assay coupled with attention to the quantity of DNA in a PCR significantly improves the detection of low-copy HPV DNA without compromising the ability of the technique to detect a wide range of HPV types or multiple infections. A touchdown approach may be especially beneficial as an analytical test for the re-evaluation of (apparently) HPV negative abnormal cervical cytological or histological samples and for investigating the association of HPV with non-anogenital lesions and tumors. Further studies are required to determine the clinical utility of a touchdown PCR approach to HPV detection and to compare the performance with recently developed (highly sensitive) multi-primer HPV PCR assays such as the PGMY [32] and the SPF_10 _[33].

## Competing interests

The author(s) declare that they have no competing interests.

## Authors' contributions

MFE conceived and designed the study, performed DNA extractions and PCR assays, and drafted the manuscript. CS-CA carried out DNA extractions, the bulk of the PCR assays, and dot blot and Southern blot hybridization analyses. LS-A performed DNA extractions, PCR, and HPV typing of VAIN tissues. KC contributed to the study conception and design.

## Pre-publication history

The pre-publication history for this paper can be accessed here:


